# Single crystal growth from separated educts and its application to lithium transition-metal oxides

**DOI:** 10.1038/srep35362

**Published:** 2016-10-17

**Authors:** F. Freund, S. C. Williams, R. D. Johnson, R. Coldea, P. Gegenwart, A. Jesche

**Affiliations:** 1 EP VI, Center for Electronic Correlations and Magnetism, Augsburg University, D-86159 Augsburg, Germany; 2 Clarendon Laboratory, University of Oxford, Parks Road, Oxford OX1 3PU, United Kingdom

## Abstract

Thorough mixing of the starting materials is the first step of a crystal growth procedure. This holds true for almost any standard technique, whereas the intentional separation of educts is considered to be restricted to a very limited number of cases. Here we show that single crystals of *α*-Li_2_IrO_3_ can be grown from separated educts in an open crucible in air. Elemental lithium and iridium are oxidized and transported over a distance of typically one centimeter. In contrast to classical vapor transport, the process is essentially isothermal and a temperature gradient of minor importance. Single crystals grow from an exposed condensation point placed in between the educts. The method has also been applied to the growth of Li_2_RuO_3_, Li_2_PtO_3_ and *β*-Li_2_IrO_3_. A successful use of this simple and low cost technique for various other materials is anticipated.

The honeycomb iridates *α*-Li_2_IrO_3_ and Na_2_IrO_3_ attracted a lot of attention after Khaliullin and co-workers proposed that these systems offer a physical realization of the Kitaev interaction^1^ in a solid^2^,^3^. Motivated by their proposal, several experimental studies on single crystalline Na_2_IrO_3_ and polycrystalline *α*-Li_2_IrO_3_ have been performed^4^,^5^,^6^. Direct evidence for the entanglement between spatial and spin directions, which is a consequence of the Kitaev exchange coupling, was recently observed in Na_2_IrO_3_ by means of diffuse magnetic X-ray scattering^7^. These experiments were facilitated by the availability of sizable single crystals^4^, however, the microscopic details of the growth are not well understood.

For *α*-Li_2_IrO_3_ it has not been possible so far to obtain single crystalline material – not even on a length scale of 10 µm. Accordingly, there has been no direct access to the anisotropy of the physical properties, and the magnetic structure as well as the contribution of the Kitaev exchange has been still under debate.

The growth procedure presented in this letter allows the growth of single crystals of *α*-Li_2_IrO_3_ of one millimeter along a side. A schematic sketch of the synthesis method and the phase formation as function of time and temperature are shown in Fig. 1. A remarkable feature is the isothermal nature of the process that was revealed by careful temperature measurements at different positions of the crucible (see Supplementary Figure 1): instead of a temperature gradient, here 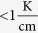, it is the formation of *α*-Li_2_IrO_3_ itself that drives the transport by maintaining a concentration gradient. The proposed, relevant transport equations are [ref. 8, p. 166, 217]:


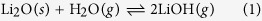



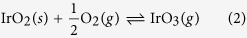


Single crystalline *α*-Li_2_IrO_3_ forms from gaseous LiOH and IrO_3_. The X-ray diffraction pattern and the sharpness of the phase transition to the magnetically ordered state revealed a superior sample quality when compared to polycrystalline material (see below). Furthermore, the magnetic structure has been solved by recent single crystal magnetic resonant X-ray diffraction measurements performed on these samples (published separately^9^). The synthesis of *α*-Li_2_IrO_3_ was first reported by Kobayashi *et al.*^10^. Polycrystalline material was obtained by heating mixtures of Li_2_CO_3_ and IrO_2_ to temperatures between 650 °C–1050 °C. The presence of a low-spin Ir^4+^ state with an effective spin 1/2, as one of the essential ingredients of the Kitaev model, was reported soon after^11^. Despite the substantial interest in this material, the basic synthesis root has not changed since: to the best of our knowledge all attempts made are based on using Li_2_CO_3_ as starting material. Heating mixtures of Li_2_CO_3_ with Ir or IrO_2_ to sufficiently high temperatures leads to the formation of *α*-Li_2_IrO_3_ under release of CO_2_. This process, often referred to as ‘calcination’, has been applied to the growth of several other related materials, e.g.: Li_2_RuO_3_^12^, Na_2_IrO_3_^13^ or Na_2_PtO_3_^13^. In this way comparatively large single crystals of Na_2_IrO_3_ were obtained^4^,^7^. The samples show a plate-like habit with typical lateral dimensions of a few square millimeter and a thickness of 100 *μ*m. They grow out of a polycrystalline base (‘poly bed’) and form predominantly at the upper part of the product. For *α*-Li_2_IrO_3_, however, the similar approach leads to only a fine powder. Different flux methods, especially pre-sintered *α*-Li_2_IrO_3_ in LiCl flux, failed to increase the crystal size. Nevertheless, a better crystallinity was inferred from X-ray powder diffraction measurements^14^. In those attempts, the LiCl does not act as a ‘classical’ flux, it rather promotes a solid state reaction with enhanced diffusion.

At temperatures above 1000 °C the formation of *α*-Li_2_IrO_3_ competes with the high-temperature polytype *β*-Li_2_IrO_3_^15^. After repetitive heating of *α*-Li_2_IrO_3_ at 1100 °C small single crystals up to several 10 μm of *β*-Li_2_IrO_3_ form^16^. Annealing *β*-Li_2_IrO_3_ at temperatures below 1000 °C did not lead to the formation of *α*-Li_2_IrO_3_, indicating that the transition is irreversible. Small single crystals of a third modification, the ‘harmonic’ honeycomb *γ*-Li_2_IrO_3_, were obtained by the calcination of Li_2_CO_3_ and IrO_2_ followed by annealing in molten LiOH at 700 °C to 800 °C^17^. An advantage of the calcination process is the ability to start from carbonates which are comparatively easy to handle and store. In contrast, elemental lithium is air sensitive and has been avoided as an educt in previous approaches. Furthermore, lithium reacts with many standard crucible materials and develops a moderately high vapor pressure (17 mbar at 900 °C^18^). On the other hand, elemental lithium has several advantages for the use as a flux. Its low melting point of 180 °C in comparison with a high boiling temperature of 1342 °C fulfill two key characteristics of a good flux^19^. Furthermore, lithium has a good solubility for iridium^20^. However, all our attempts to grow single crystals of *α*-Li_2_IrO_3_ from a lithium-rich flux and mixtures of lithium with LiCl, LiOH, LiBO_2_ and/or Li_2_CO_3_ failed. A comprehensive overview of those attempts is given in the Supplementary Table 1.

Comparatively large single crystals of several millimeter along a side, as observed for Na_2_IrO_3_^4^,^7^ are not expected to grow in a solid state reaction due to the limited diffusion length. Given that the calcination process is completed at these temperatures (at 1050 °C) and the compound does not melt congruently indicates the relevance of a vapor transport process within the crucible. In order to investigate the possible formation and transport of Li-O, Ir-O, and/or Li-Ir-O gas specious during the syntheses of *α*-Li_2_IrO_3_, we started a growth attempt from elemental lithium and iridium in air. Lithium granules were placed on iridium powder in an Al_2_O_3_ crucible. The mixture was heated to 900 °C over 4 h, held for 72 h and quenched to room temperature. To our surprise, already the first attempt revealed *α*-Li_2_IrO_3_ single crystals of up to 50 μm along a side. The whole product appeared homogeneous and X-ray powder diffraction pattern showed only small amounts of IrO_2_ and Ir. This is even more surprising since only three small lithium granules (roughly 4 mm in length with a diameter of 1.5 mm) were used but *α*-Li_2_IrO_3_ formed over the whole bottom of the crucible (inner diameter of 16 mm). This observation strongly supports the idea of vapor transport playing a decisive role for the growth of this material. However, a classical vapor transport along a temperature gradient does not seem to take place: various growth attempts in a horizontal tube furnace indicated that once *α*-Li_2_IrO_3_ has formed it does not transport anymore. This observation is corroborated by an estimate of the free enthalpy of formation for *α*-Li_2_IrO_3_: 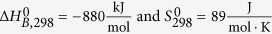 [M. Schmidt, MPI-CPfS, private communication]. It corresponds to a large, exothermic value of the free reaction enthalpy of 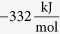 for the following equilibrium equation:


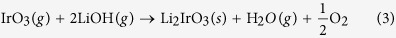


Therefore, we started to investigate the growth from spatially separated educts. For this purpose a specially designed setup has been constructed as depicted in Fig. 2a–c. It consists of a standard crucible, rings (washers), rings with spikes and a disc with a center hole (aperture). All parts are made from Al_2_O_3_. The rings act as spacers, hold the aperture in place and allow to vary the distance between starting materials and spikes.

The spikes provide an exposed condensation point in between the educts. They are stacked as a ‘spiral staircase’ in order to identify the ideal position for the growth. The aperture is placed above the spikes and acts as a platform for one of the starting materials. The other educt is placed on the bottom of the crucible in the center of two spacers. This avoids a direct contact between the material and the spikes which sit on top of the spacers. For the growth of *α*-Li_2_IrO_3_, iridium metal powder and lithium granules are used as starting materials. Iridium is placed on the bottom of the crucible, lithium on top of the aperture. The distance between the elements is roughly 11 mm with five spikes placed in-between. The masses were chosen in their stoichiometric ratio. The whole setup is placed in a box furnace at 200 °C, heated to 1020 °C with a rate of 180 °C per hour, held for three days and finally quenched to room temperature. While heating, lithium transforms to a Li_2_O/LiOH mixture at moderate temperatures. At 900 °C all lithium is burned to Li_2_O. See a detailed analysis of this process in the Supplementary Figure 2. Only small amounts of Li_2_O are found on top of the aperture (where the lithium was placed) after the process. The iridium powder placed at the bottom is partially oxidized to IrO_2_. *α*-Li_2_IrO_3_ covers large parts of the spikes, with the largest crystals growing at the tip of the spikes, 3–4 mm above the iridium (Fig. 2d).

Single crystals of dimensions larger than 1 mm were obtained (Fig. 3a). A good sample quality is inferred from Laue-back-reflection (Fig. 3b): the diffraction pattern shows the (nearly) three-fold rotational symmetry of the honeycomb layers (along the c*-direction). The spot-size of the X-ray beam was similar to the sample dimensions. Figure 3c shows the temperature dependent specific heat measured on the same single crystal in comparison with polycrystalline material that was grown by calcination^5^. The improved sample quality of the single crystal is apparent from a sharper transition to the antiferromagnetically ordered state at *T*_N_ = 15 K. Temperature-dependent magnetic susceptibility for field applied parallel (*χ*_*ab*_) and perpendicular (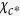) to the honeycomb layers is shown in Fig. 3d. An easy-plane behavior with a sharp decrease of *χ*_*ab*_ at *T*_N_ is observed, whereas 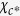 decreases only slightly. A measurement performed on polycrystalline material^5^ is included for comparison and can be roughly described by 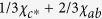 for *T* > *T*_N_.

The structural order in the grown crystals was probed using X-ray diffraction and representative patterns are shown in Fig. 4a–d. The data are fully consistent with the expected monoclinic crystal structure^21^ of alternate stacking of honeycomb Li_1/2_IrO_3_ and hexagonal Li layers with space group *C*2/*m* (calculated patterns shown in Fig. 4e–f), and details of the full structural refinement are given in the Supplementary Note. Samples grown at 900 °C showed pronounced rods of diffuse scattering along the c* direction, normal to the layers, as evidenced in Fig. 4c. Rods of diffuse scattering with the same selection rule were also observed in the iso-structural materials Na_2_IrO_3_ and *α*-RuCl_3_^22^ and attributed^23^ to occasional in-plane shifts of the stacked layers by ±**b**/3. Monitoring the structural order for different growth temperatures allowed us to optimize the synthesis parameters and obtain crystals with almost no detectable diffuse scattering (compare Fig. 4bc, and the intensity profile in Fig. 4g), showing that those crystals grown at 1020 °C are close to the limit of fully-coherent, three-dimensional structural ordering. Figure 4a,b show data from an un-twinned single crystal. Most as-grown crystals are twinned and a representative diffraction pattern shown in Fig. 4d can be understood by three additional twins: two twins rotated by ±120° around c*, and another twin with the **a** and **c** axes interchanged (for more details see Supplementary Note). We note that the susceptibility data in Fig. 3d was collected on a crystal that contained predominantly twins rotated by ±120° around c*, so under the assumption that the susceptibility tensor has only one unique axis c* (normal to the *ab* plane), all those twins had the same magnetic response in field applied along c* or perpendicular.

The method described is not restricted to the growth of *α*-Li_2_IrO_3_. Single crystals of *β*-Li_2_IrO_3_ and Li_2_RuO_3_ were also obtained (Fig. 5a,b, see Supplementary Figure 3 for X-ray diffraction pattern). Formation of the latter is expected from the similar transport behavior of Ir and Ru [ref. 8, p. 214 ff]. For Li_2_PtO_3_ we obtained polycrystalline material of good quality (Fig. 5c, see Supplementary Figure 3 for X-ray diffraction pattern). In conclusion, the technique should be applicable to various transport active elements in air in its simplest form. Application to a broader class of materials could be achieved by providing a controlled atmosphere (static or flowing) of, for example, oxygen, chlorine or iodine. The combination of an isothermal vapor transport in an open crucible with separated educts is unique and provides another approach for the crystal growth community.

## Additional Information

**How to cite this article**: Freund, F. *et al.* Single crystal growth from separated educts and its application to lithium transition-metal oxides. *Sci. Rep.* **6**, 35362; doi: 10.1038/srep35362 (2016).

## Supplementary Material

Supplementary Information

## Figures and Tables

**Figure 1:**
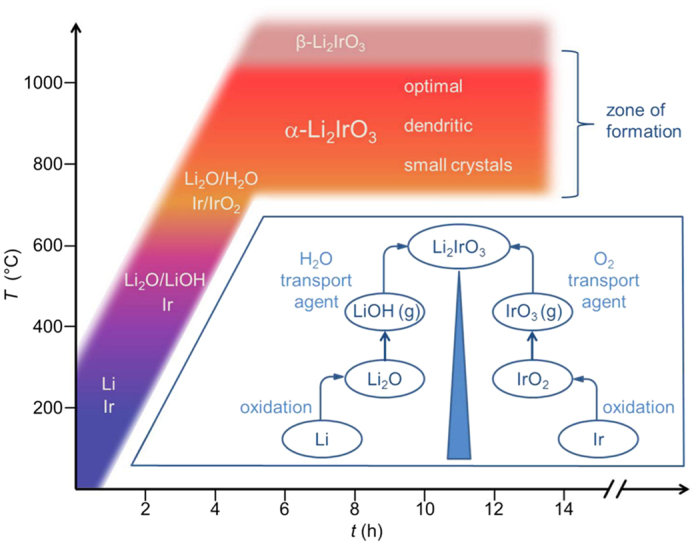
Schematic description of the synthesis method (inset) and temperature profile. Elemental Li and Ir are spatially separated in an open crucible. Upon heating in air Li initially forms solid LiOH that transforms to Li_2_O at *T* > 500 °C. Ir partially oxidizes to IrO_2_. The formation of *α*-Li_2_IrO_3_ takes place at *T* = 750–1050 °C. Single crystals grow from an exposed condensation point placed in between the educts.

**Figure 2:**
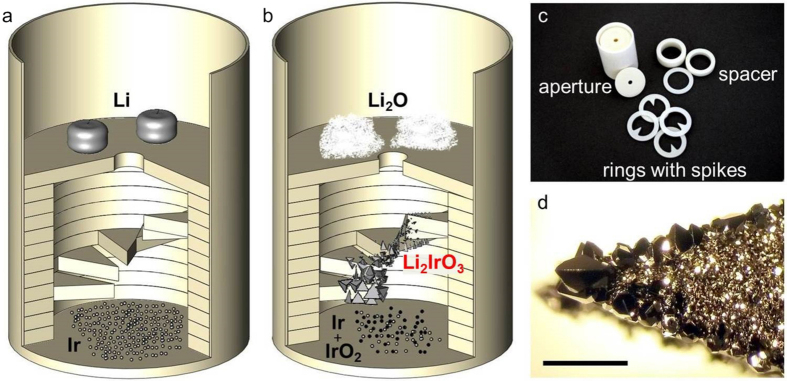
Crystal growth equipment (crucible diameter 16 mm). Arrangement of the materials before and after the growth process is depicted in (**a,b**), respectively. The rings with spikes are oriented like a spiral staircase in order to allow for nucleation at different positions with less intergrowth of the crystals. Formation of the largest *α*-Li_2_IrO_3_ single crystals is observed on spikes placed roughly 4 mm above the Ir starting material. (**c**) individual setup parts made from Al_2_O_3_ and (**d**) typical appearance of one of the lower spikes covered with *α*-Li_2_IrO_3_ crystals at the bottom side, scale bar 1 mm.

**Figure 3:**
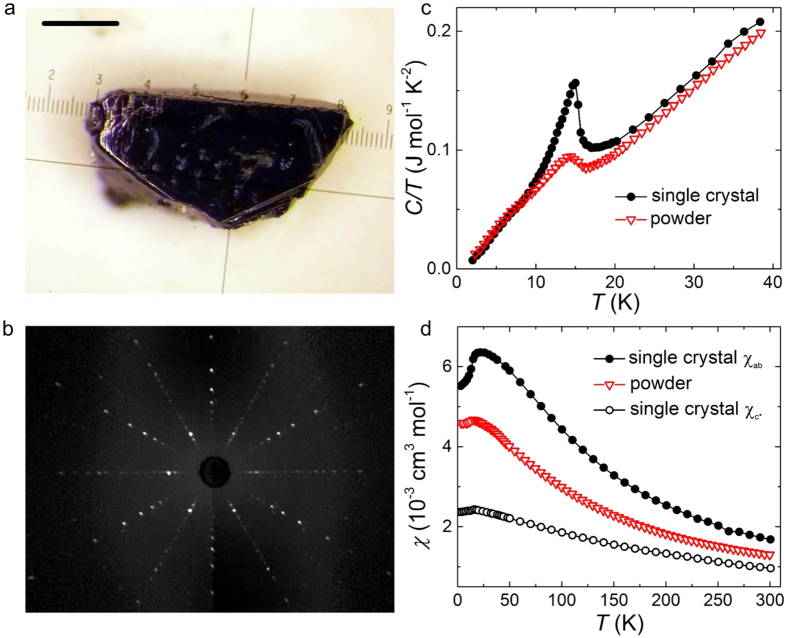
Sample quality and magnetic anisotropy of *α*-Li_2_IrO_3_. (**a**) comparatively large single crystal (1.2 mm × 0.4 mm × 0.5 mm and *m* = 1.7 mg) grown from separated educts (scale bar 0.3 mm). The corresponding Laue-back-reflection pattern, depicted in (**b**), shows the (nearly) three-fold rotation symmetry perpendicular to the honeycomb layers. (**c**) temperature dependent specific heat of the single crystal shown in (**a**) in comparison with a typical polycrystalline sample. (**d**) an easy-plane anisotropy is apparent from the temperature dependent magnetic susceptibility (*μ*_0_*H* = 1 T, *χ*_*ab*_ : *H*⊥c^*^, 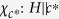).

**Figure 4:**
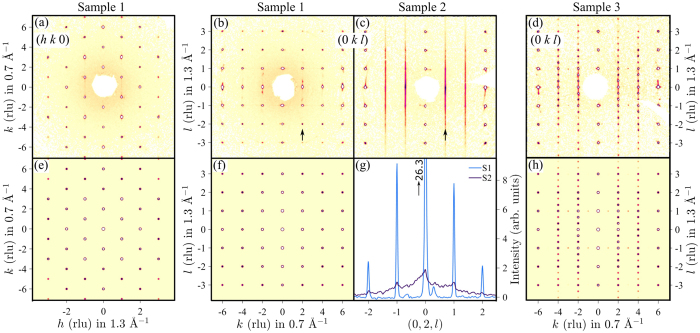
X-ray diffraction pattern from three different *α*-Li_2_IrO_3_ crystals: one un-twinned and without stacking faults (sample 1, panels (**a**,**b**)), one predominantly a single grain, but with significant stacking faults manifested in extended diffuse scattering along *l* (sample 2, panel **c**), and one multi-twin crystal (sample 3, panel **d**). Color is intensity on a log scale. Vertical arrows near *k* = 2 in panels **b**,**c** show direction along which the intensity is plotted in panel **g**, note the strong contrast between sample 1 with sharp peaks at integer *l* and sample 2 where diffuse scattering dominates. Bottom graphs (**e,f,h**) show the calculated X-ray diffraction pattern in the same axes as the above panels **a**,**b**,**d**, for the nominal monoclinic crystal structure of *α*-Li_2_IrO_3_^21^. Panel **h** includes contribution from C^±^ twins (grains rotated by ±120° around c* leading to the peaks at fractional coordinates (0, *k*, *n* ± 1/3), with *n* integer and *k* = ±2, ±4) and an A-type twin responsible for the peaks at (0, *k*, ±1) with *k* odd (see Supplementary Note for details).

**Figure 5:**
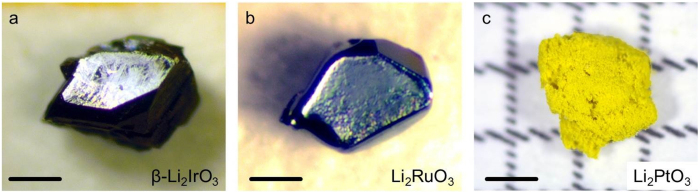
Further materials synthesized from separated educts. (**a**) *β*-Li_2_IrO_3_ single crystal (scale bar 0.2 mm) and (**b**) a single crystal of Li_2_RuO_3_ (scale bar 0.1 mm). For Li_2_PtO_3_ fine yellow powder could be obtained shown in (**c**) (scale bar 1 mm).
