# Supplementary material for: Single crystal growth from separated educts and its application to lithium transition-metal oxides
Source: Sci Rep. 2016 Oct 17;6:35362. doi: 10.1038/srep35362 (PMC5066249; doi:10.1038/srep35362)
Supplement: Supplementary Information [file srep35362-s1.pdf]

Supplementary Information to ”Single crystal growth from separated educts and its application to lithium transition-metal oxides”

F. Freund[1], S. C. Williams[2], R. D. Johnson[2], R. Coldea[2], P. Gegenwart[1], and A. Jesche[1,*]

[1]EP VI, Center for Electronic Correlations and Magnetism, Augsburg University, D-86159 Augsburg, Germany
[2]Clarendon Laboratory, University of Oxford, Parks Road, Oxford OX1 3PU, United Kingdom
[*]anton.jesche@physik.uni-augsburg.de

Supplementary Table 1. Results of various attempts to grow α-Li_2IrO_3 single crystals from flux.

Flux	Starting Material	Crucible	Temperature Profile	Result
Li	α-Li_2IrO_3	Ta	4 h to 1000 °C	crucible decomposed
Li	α-Li_2IrO_3	Nb	4 h to 1000 °C	crucible decomposed
Li	IrO_2	Nb	5 h to 1000 °C; hold 5 h; 60 h to 200 °C	Ir single crystals, crucible attacked
Li, $LiBO_2$	α-Li_2IrO_3	Pt in quartz	5 h to 950 °C; hold 1 h; 60 h to 500 °C	quartz tube broken, α-Li_2IrO_3 transformed to IrO_2
LiCl, Li_2CO_3	α-Li_2IrO_3	Al_2O_3 in quartz	2 h to 1000 °C; hold 2 h; 55 h to 500 °C	well ordered α-Li_2IrO_3 powder, β-Li_2IrO_3 impurities
LiCl, LiOH	α-Li_2IrO_3	Al_2O_3 in quartz	2 h to 1000 °C; hold 2 h; 55 h to 500 °C	well ordered α-Li_2IrO_3, β-Li_2IrO_3 and IrO_2
LiCl, Li_2CO_3	α-Li_2IrO_3	Al_2O_3 in quartz	2 h to 850 °C; hold 2 h; 55 h to 500 °C	α-Li_2IrO_3 pellet not solved in flux
LiCl, LiOH	α-Li_2IrO_3	Al_2O_3 in quartz	2 h to 850 °C; hold 2 h; 55 h to 500 °C	α-Li_2IrO_3 powder
LiOH	α-Li_2IrO_3	Al_2O_3 in Nb	8 h to 800 °C; hold 6 h; 50 h to 500 °C	α-Li_2IrO_3 powder, Ir impurities
LiCl	α-Li_2IrO_3	Al_2O_3 in quartz	2 h to 850 °C; hold 2 h; 55 h to 500 °C	α-Li_2IrO_3 powder, β-Li_2IrO_3 and IrO_2 impurities

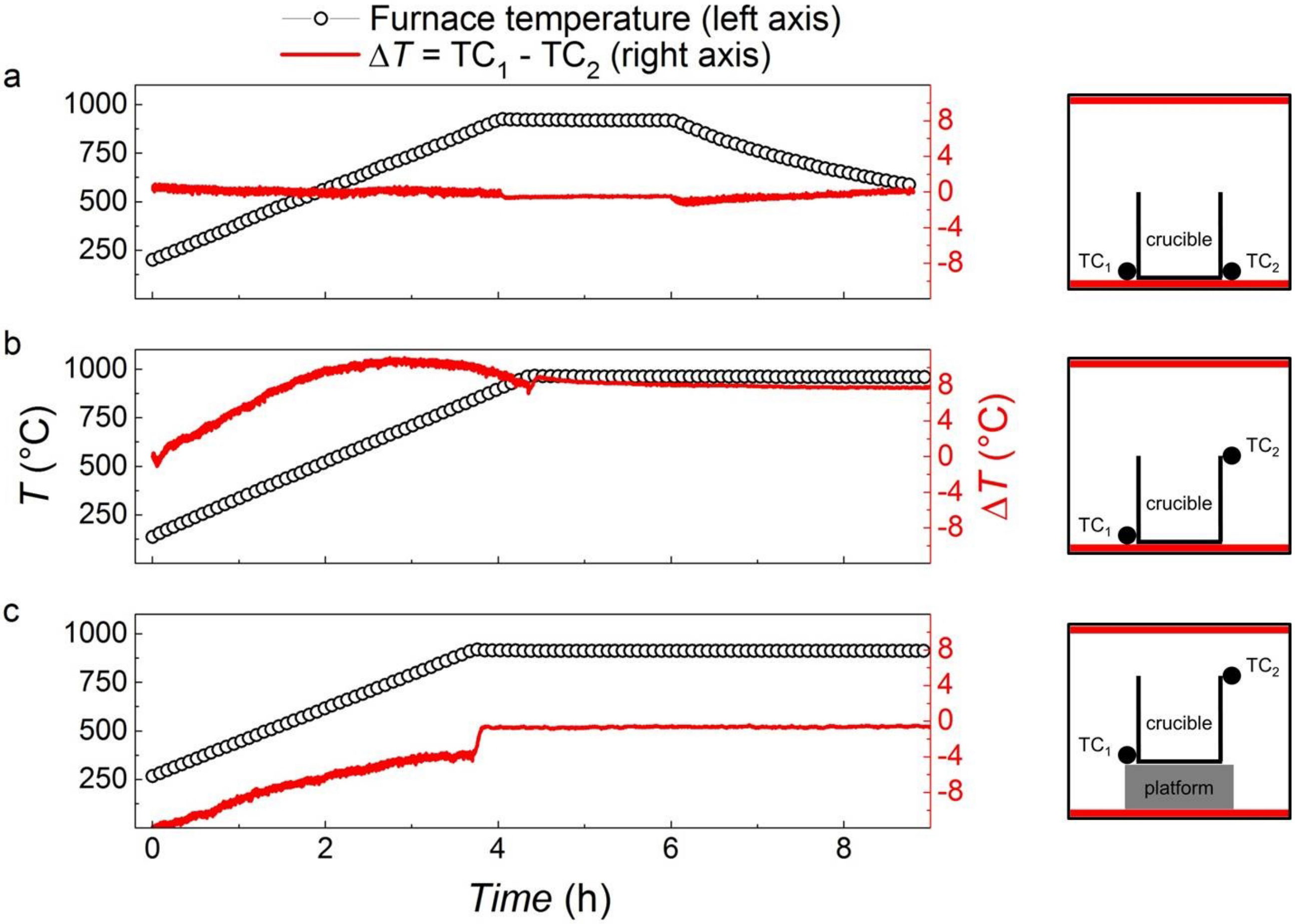

Supplementary Figure 1. Temperature gradient between bottom and top of the crucible during the growth. Temperature profiles are shown on the left hand side. The position of the thermocouples (TCs) are depicted on the right. The heating plates of the furnace are placed on the top and bottom (marked in red). a), verification of the calibration of the used thermocouples. TC_1 and TC_2 are both placed on the bottom of the crucible. The crucible stands on the lower heating plate. A small difference in the range of $\Delta T \approx \pm 1\,^{\circ}\mathrm{C}$ is confirmed. b), TC_1 stays at the bottom and TC_2 is placed on the top. The gradient increases during the heating process. At the final temperature the difference is constant and small with $\frac{\Delta T}{\Delta z} \approx \frac{8\,\mathrm{K}}{2\,\mathrm{cm}} = 4\frac{\mathrm{K}}{\mathrm{cm}}$. c), in order to investigate the effect of the small temperature gradient observed in b), the whole setup is placed on a platform to equalize the distance of the crucible to the upper and lower heating plate of the furnace. As expected for a symmetric arrangement $\Delta T \approx 0\,\mathrm{K}$. The growth is not affected and it is still possible to grow α-Li_2IrO_3 single crystals in this configuration. This shows the minor importance of a temperature gradient in this method.

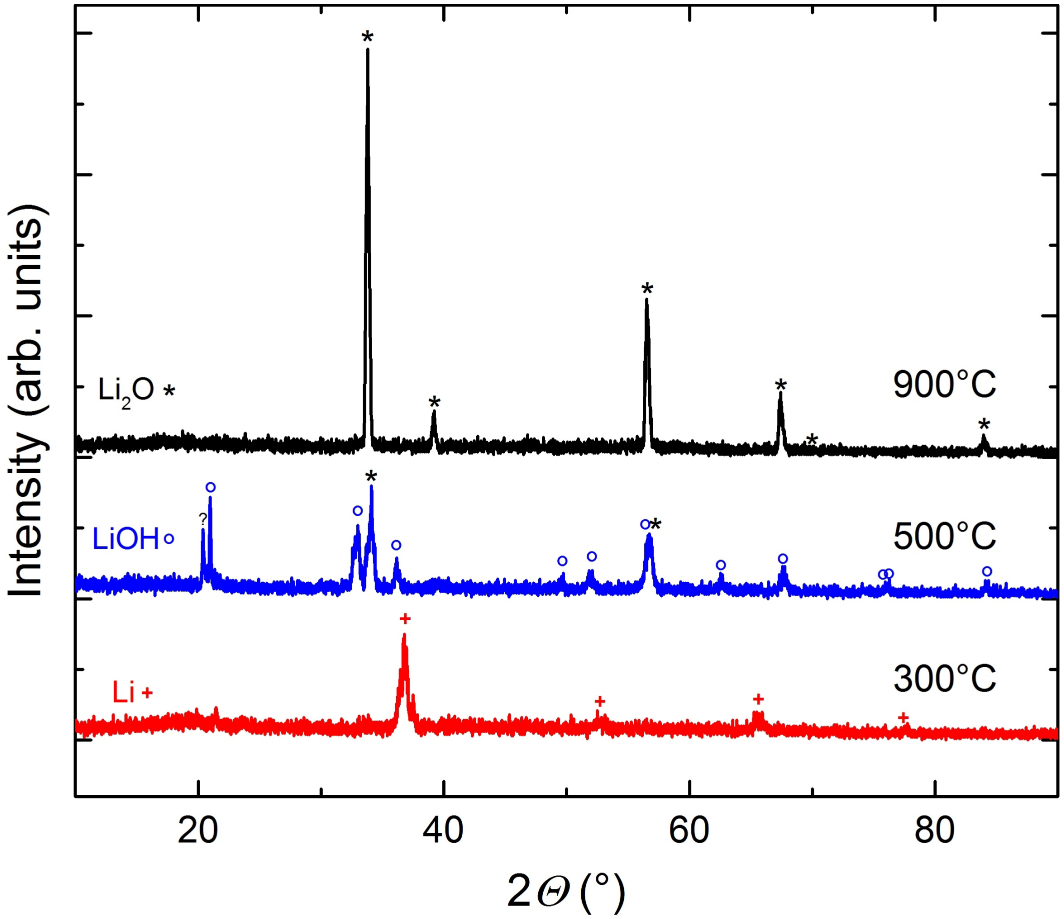

Supplementary Figure 2. Evolution of the lithium educt during the heating process. Three pieces of elemental lithium are placed in three alumina crucibles, put in an open box furnace at 200 °C and heated to 900 °C in four hours. The samples are taken out of the furnace during heating at 300 °C, 500 °C and 900 °C, respectively. Powder X-ray diffraction patterns, collected immediately after taking out the sample, show the transformation of lithium to a Li_2O-LiOH mix, ending in pure Li_2O. The theoretical peak positions of Li, Li_2O and LiOH are indicated in the patterns.

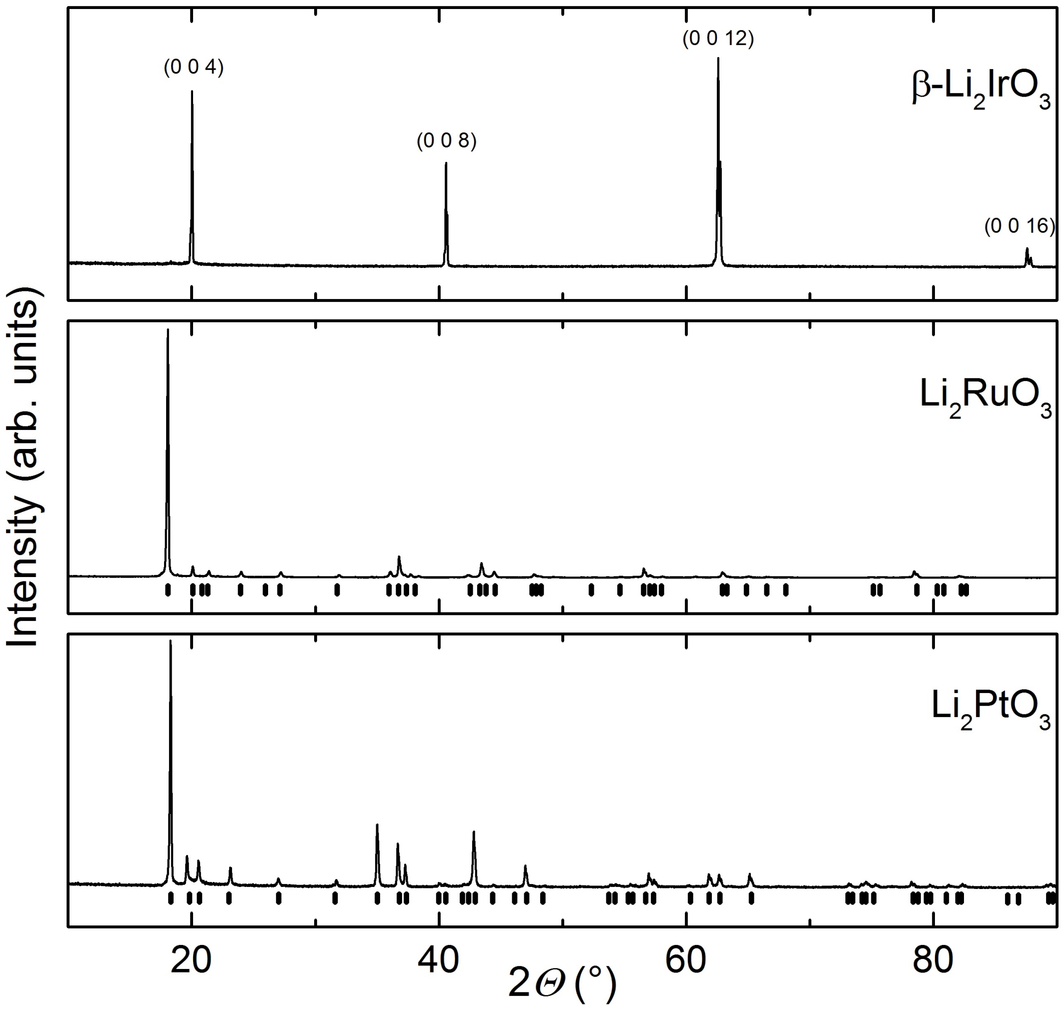

Supplementary Figure 3. X-ray diffraction patterns of β-Li_2IrO_3, Li_2PtO_3 and Li_2RuO_3 using a Rigaku powder diffractometer. A single crystal of β-Li_2IrO_3 was aligned with the (00l) plane satisfying the Bragg's law. For Li_2RuO_3 single crystals were crushed, while Li_2PtO_3 crystallized in well ordered powder. The samples are phase pure and all peaks can be indexed based on the reported crystal structures (black bars below the diffraction patterns). All samples are synthesized using separated educts (Li, Ir/Ru/Pt).

Supplementary Note

Crystal Structure of α-Li_2IrO_3

The crystal structure of α-Li_2IrO_3 is shown in Suppl. Fig. 4 and consists of an alternate stacking of honeycombs of edge-sharing IrO_6 octahedra with Li in the center, and hexagonal Li layers. Adjacent layers are stacked with an in-plane offset, leading to a monoclinic $C2/m$ space group[1]. Diffraction data were collected on 'Sample 1' (samples are described in the main text, alongside representative data, shown in Fig 4.a-b). The diffraction pattern could be fully indexed with a monoclinic unit cell with lattice parameters (Supplementary Table 2) consistent with earlier reports[1], with the empirical selection rules for the observed Bragg peaks $h+k=$ even, as expected for a C-centered cell in the ab plane. Importantly, this sample showed no observable diffuse scattering, which would constitute qualitative evidence for stacking faults, nor was there evidence for twinning (discussed below). This allowed for a single phase structural model to be considered without introduction of twin models or site-mixing approximations for disorder.

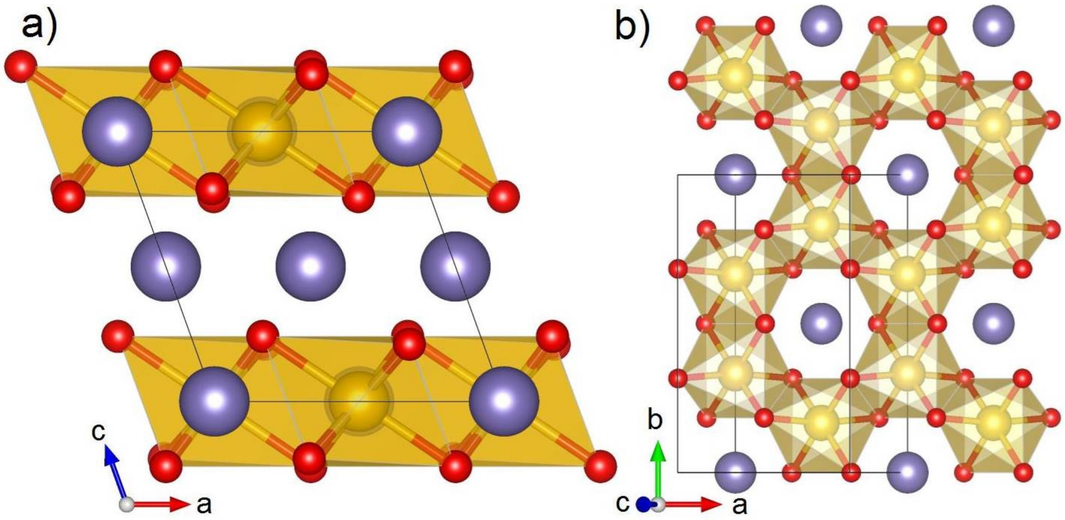

Supplementary Figure 4. Refined crystal structure of α-Li_2IrO_3, showing unit cell as a black outline, Ir as gold spheres, Li as purple and O as red. a) Projection onto the ac plane, showing the alternate stacking of $Li_{1/2}IrO_3$ and Li layers. b) Basal plane layer showing the honeycomb of edge-sharing IrO_6 octahedra.

We performed a full structural refinement of the crystal structure parameters against all integrated X-ray diffraction intensities. The refinement gave a good agreement between model and data as shown in Suppl. Fig. 5, with reliability factors $R(F^2) = 9.83\%$, $wR(F^2) = 14.6\%$, $R(F) = 5.82\%$ and $\chi^2 = 3.82$. The refined room temperature crystallographic parameters and quantitative details of the data collection are given in Supplementary Table 2, and the corresponding structure is drawn in Suppl. Fig. 4. In addition, we tested whether or not the crystal structure had lower symmetry than $C2/m$ by describing it within the corresponding $P\bar{1}$ primitive unit cell. This unit cell preserves all translational symmetry of the lattice, but

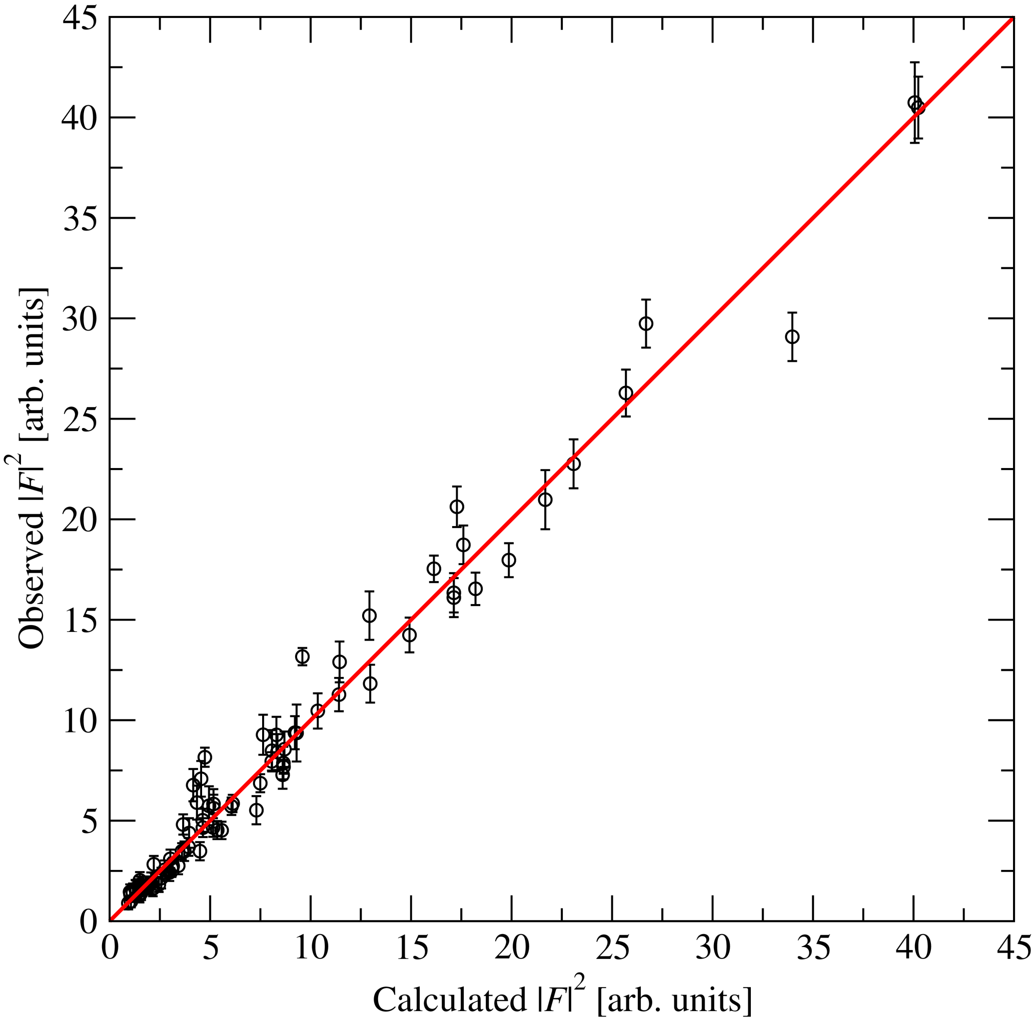

Supplementary Figure 5. Observed vs calculated $|F|^2$ values from Rietveld refinement of the $C2/m$ crystal structure against the single crystal diffraction data. The red line is a guide to the eye.

only contains inversion symmetry. In this case, no improvement in the fit was achieved, and the same result was found within error as for the higher-symmetry, nominal $C2/m$ cell in Supplementary Table 2. Further tests to explore Ir disorder through site-mixing with the Li ions also showed no improvement in the refinement, leading us to the conclusion that Sample 1 has a fully ordered, layered iridium honeycomb lattice in all three dimensions. We note that a key difference between the solution presented herein and that published by O'Malley *et al*. is the z-coordinate of O2. In our solution, the oxygen ions lie more closely within a common ab-plane, as observed in Na_2IrO_3[2].

Supplementary Table 2. α-Li_2IrO_3 room temperature crystal structure. Standard errors on refined parameters are given in parenthesis. Note that lithium parameters were fixed to literature values[1].

Cell parameters

Space group: $C2/m$

Z = 4

a,b,c (Å)	5.1749(2)	8.9359(2)	5.1194(2)
α,β,γ (°)	90	109.827(5)	90

Volume: 222.70(2) $Å^3$

Atomic fractional coordinates

Atom	x	y	z	U_{iso}($Å^2$)
Ir1	0	0.3331(4)	0	-
Li1	0	0	0	0.00633
Li2	0	0.809	0.5	0.00633
Li3	0	0.5	0.5	0.00633
O1	0.25(1)	0.321(5)	0.765(8)	0.03(1)
O2	0.27(2)	0	0.76(1)	0.03(1)

Ir anisotropic adps ($Å^2$)

U_{11}	0.019(7)
U_{22}	0.007(2)
U_{33}	0.028(3)
U_{12}	-
U_{13}	0.011(3)
U_{23}	-

Data collection

measured reflections: 2415

independent reflections: 83

Data reduction R_{int}: 5.76%

(Criterion for observed reflections: $I > 2.0\sigma(I)$)

observed reflections: 83

fitted parameters: 12

Stacking Faults in α-Li_2IrO_3

As discussed in detail in the case of the iso-structural Na_2IrO_3[2], as a result of the near hexagonal symmetry of the honeycomb layers there is only a rather small energy cost associated with a honeycomb layer being displaced in-plane by $\pm\mathbf{b}/3$. The presence of such occasional in-plane displacements in the layer stacking sequence is manifested in the X-ray diffraction pattern by diffuse rods of scattering along the l direction with the selection rule $k = 3n+1$ or $3n+2$ (n integer) and $h+k$ = even (due to the C-centering). The diffraction patterns of two samples affected by stacking faults to different extents are compared in Fig. 4b−c in the main text and in the intensity profile in Fig. 4g in the main text. It is clear that while sample 1 shows very strong peaks at integer l with negligible diffuse signal in-between the sharp peaks, by contrast for sample 2 the Bragg peaks are very weak relative to the strong diffuse scattering, which extends across a wide range of l values.

Twinning in α-Li_2IrO_3

There is a high incidence of twinning in α-Li_2IrO_3 crystals. Most common are twins with the ab planes rotated relative to one another by 120° around the normal direction, $\mathbf{c}^*$. The occurrence of such twins can be understood as follows. In the nominal structure subsequent honeycomb layers are vertically stacked with an in-plane offset along the $-\mathbf{a}$ direction, see Supplementary Figure 4a. Since the honeycomb layers have a near three-fold rotational symmetry around the vertical axis, there would be only a small energy cost if the in-plane offset direction were to rotate in the ab plane by ±120°, and, if subsequent layers were then to stack according to this new offset direction, a new grain would form, rotated around the $\mathbf{c}^*$ direction of the primary grain. We label such a twin $C^{\pm}$, where the superscript indicates the sign of the ±120° rotation.

Another common twin (designated as type A) corresponds to a rotation by 180° around the (101) direction, which swaps the $\mathbf{a}$ and $\mathbf{c}$ axes. This might occur because the a and c lattice parameters are very similar in magnitude. A third type of twin found corresponds to a rotation by ±90° around the $(\bar{1}01)$ axis, designated as $B^{\pm}$. We note that for $a = c$ an A-type rotation is equivalent to two consecutive B-type rotations followed by a 180° rotation around (010), i.e. $A = 2_y(B^{\pm})^2$. For the given crystal structure the two-fold rotation around $\mathbf{b}$ leaves the atomic arrangement invariant as it is a symmetry operation of the $C2/m$ space group. The above twinning types are summarized in Supplementary Table 3, where the last column gives the transformation matrix between the reciprocal space coordinates (h', k', l') of the rotated twin and the corresponding coordinates of the primary (un-rotated) twin, defined as

$$(hkl) = (h'k'l')\mathscr{M}. \tag{1}$$

For simplicity the transformation matrix is given in the case when the structure has a hexagonal metric, where $a : b : c = 1 : \sqrt{3} : 1$ and $\cos\beta = -1/3$ (the latter equation corresponds to eclipsed stacking at the third layer). Those conditions are satisfied in the actual crystal structure to better than 1.4%.

Supplementary Table 3. Common twinning types in α-Li_2IrO_3 crystals. Last column gives the transformation matrix $\mathscr{M}$ for the reciprocal space coordinates as defined in (1).

Type	Orientation	$\mathscr{M}$
A	rotation by 180° around (101), exchanges **a** and **c**.	$\begin{pmatrix} 0 & 0 & 1 \\ 0 & -1 & 0 \\ 1 & 0 & 0 \end{pmatrix}$
$B^{\pm}$	rotation by $\pm 90°$ around $(\bar{1}01)$, brings the $\pm\mathbf{b}^*$ axis along (101)	$\begin{pmatrix} 1/2 & \pm 3/2 & -1/2 \\ \mp 1/3 & 0 & \mp 1/3 \\ -1/2 & \pm 3/2 & 1/2 \end{pmatrix}$
$C^{\pm}$	rotation by $\pm 120°$around (001)	$\begin{pmatrix} -1/2 & \pm 3/2 & 1/2 \\ \mp 1/2 & -1/2 & \pm 1/6 \\ 0 & 0 & 1 \end{pmatrix}$

Fig. 4d in the main text shows the diffraction pattern for a multi-twinned crystal. Using the transformation matrices for the various types of twins we identify the peaks near fractional positions $(0,2,n+1/3)$ (n integer) as being the nominal $(1\bar{1}n)$ reflections of a C^+ twin, whereas peaks near $(0,2,n-1/3)$ are the nominal $(\bar{1}\bar{1}n)$ reflections of a C^- twin. Similarly, the peaks near $(0,k,\pm 1)$, k = odd are identified as being the nominal $(\pm 1,-k,0)$ reflections of an A twin. All peaks seen in the diffraction pattern can then be accounted for by an appropriate weighting of a primary grain with A, C^+ and C^- twins, compare Fig. 4d and h) in the main text. The presence of $B^{\pm}$ twins is most easily revealed by the diffraction pattern in the $(h0l)$ plane (not shown) via peaks at $(2n/3,0,2n/3)$ (n integer), which correspond to nominal $(0,\mp 2n,0)$ reflections of the rotated grain. The B^+ or B^- twins can subsequently be distinguished for example by the diffraction pattern in the $(\bar{h}kh)$ plane (not shown) where the nominal (001) reflection of the twins will appear at $(-1/2,\pm 3/2,1/2)$, respectively. We note that all the above types of twins could also occur in combination, i.e. an A type twin of the primary grain may have its own $C^{\pm}$ twins and so on.

References

1. O'Malley, M. J., Verweij, H. & Woodward, P. M. Structure and properties of ordered Li_2IrO_3 and Li_2PtO_3. *J. Solid State Chem.* **181**, 1803 – 1809 (2008)

2. Choi, S. K. *et al.* Spin waves and revised crystal structure of honeycomb iridate Na_2IrO_3. *Phys. Rev. Lett.* **108**, 127204 (2012).
